# Monitoring Cellular Movement with Photoconvertible Fluorescent Protein and Single-Cell RNA Sequencing Reveals Cutaneous Group 2 Innate Lymphoid Cell Subtypes, Circulating ILC2 and Skin-Resident ILC2

**DOI:** 10.1016/j.xjidi.2021.100035

**Published:** 2021-07-02

**Authors:** Minori Nakatani-Kusakabe, Koubun Yasuda, Michio Tomura, Makoto Nagai, Kiyofumi Yamanishi, Etsushi Kuroda, Nobuo Kanazawa, Yasutomo Imai

**Affiliations:** 1 Department of Dermatology, Hyogo College of Medicine, Nishinomiya, Japan; 2 Department of Immunology, Hyogo College of Medicine, Nishinomiya, Japan; 3 Laboratory of Immunology, Faculty of Pharmacy, Osaka Ohtani University, Osaka, Japan

**Keywords:** AD, atopic dermatitis, DC, dendritic cell, dLN, draining lymph node, ILC2, group 2 innate lymphoid cell, KikGR, Kikume Green-Red, MHC, major histocompatibility complex, RNA-seq, RNA sequencing

## Abstract

We previously generated a transgenic mouse line expressing skin-specific IL-33 (IL33tg mice) and showed that IL-33 elicits group 2 innate lymphoid cell (ILC2)–dependent atopic dermatitis–like skin inflammation. ILC2s are believed to be tissue-resident cells under steady-state conditions, but the dynamics of ILC2 migration are not fully understood. We sorted ILC2s from the skin and draining lymph nodes of IL33tg mice and analyzed their transcriptomes using the single-cell RNA sequencing technique, which revealed that the skin ILC2s had split into two clusters: circulating ILC2 and skin-resident ILC2. The circulating ILC2s expressed H2-related major histocompatibility complex class II genes. Conversely, the skin-resident ILC2s demonstrated increased mRNA expression of the ICOS, IL-5, and IL-13. Next, we tracked ILC2 migration using IL33tg–Kikume Green-Red mice. Exposing the IL33tg–Kikume Green-Red mice’s inflamed skin to violet light allowed us to label the circulating ILC2s in their skin and track the ILC2 migration from the skin to the draining lymph nodes. Cutaneous local innate responses could transition to systemic type 2 responses by migrating the activated ILC2s from the skin into the draining lymph node. Conversely, the skin-resident ILC2s produced a large number of cytokines. Thus, the skin ILC2s turned out to be a heterogeneous cell population.

## Introduction

There are two types of immune responses: nonantigen-specific innate immunity and antigen-specific acquired immunity. Generally, antigens such as house dust mites are necessary to activate acquired immunity, but they are not necessary to activate innate immunity. IL-33 (Imai, 2019; Schmitz et al., 2005) is a representative proinflammatory cytokine of the innate immune system. Furthermore, it can induce type 2 cytokines such as IL-5 and IL-13 by directly activating innate immune system cells such as group 2 innate lymphoid cells (ILC2s) without antigen stimulation (Kim, 2015; Kobayashi et al., 2020; Moro et al., 2010). In humans, ILC2 is known to infiltrate the dermatitis lesions of patients with atopic dermatitis (AD) (Brüggen et al., 2016; Kim et al., 2013). In addition, increased frequency of circulating ILC2s in AD has been reported (Mashiko et al., 2017), and patients with AD who have a high percentage of ILC2s in their peripheral blood have been reported to respond better to IL-4/13 inhibitors such as dupilumab (Imai et al., 2021). Thus, it is already known that ILC2 is deeply involved in the pathogenesis of AD.

IL-33 is highly expressed in the epidermal keratinocytes in AD (Savinko et al., 2012), and IL-33 expression in the epidermis is known to correlate with the severity of skin rashes as indicated by SCORing Atopic Dermatitis and Eczema Area and Severity Index (Guttman-Yassky et al., 2019). To reveal the role of IL-33 in cutaneous inflammation, we previously generated transgenic mice expressing IL-33 (IL33tg) driven by a keratin 14 promoter (Imai et al., 2013). The IL33tg mice spontaneously developed AD-like skin inflammation, and the ILC2s, which produce IL-5 and IL-13, were significantly increased in the lesional skin and draining lymph nodes (dLNs). Therefore, it was found that an excess of IL-33 causes AD. Notably, the AD-like dermatitis in the IL33tg mice was ILC2 dependent (Imai et al., 2019) because the development of the dermatitis was completely suppressed when the ILC2s were depleted. However, the subtype of ILC2s in the skin remained unknown. It was expected that the ILC2s in the skin would increase because IL-33 was overexpressed in the skin of the IL33tg mouse. However, an unexpected increase in ILC2 was observed in the dLNs in this mouse because IL-33 is a cytokine that acts locally and is degraded as soon as it is secreted outside the cell (Cayrol et al., 2018). Moreover, IL-33 cannot be detected even in the blood of IL33tg mice (Imai et al., 2013). Therefore, contrary to the existing reports that ILC2 does not migrate at homeostasis (Dutton et al., 2019; Moro et al., 2016), we considered the possibility that ILC2 migrates from the skin to the lymph nodes when severe dermatitis occurs. In this study, we conducted single-cell profiling of ILC2s from the skin and dLNs from an IL33tg mouse, defining circulating and skin-resident ILC2s. We also showed that ILC2 migrates from the skin to the dLNs during inflammation using Kikume Green-Red (KikGR) knock-in mice (Tomura et al., 2014). We labeled the ILC2s within the mice’s skin KikGR-Red^+^, thus quantifying the cutaneous ILC2 dynamics.

## Results

### Splitting of skin ILC2 into two clusters as revealed by single-cell RNA sequencing

**Figure 1:**
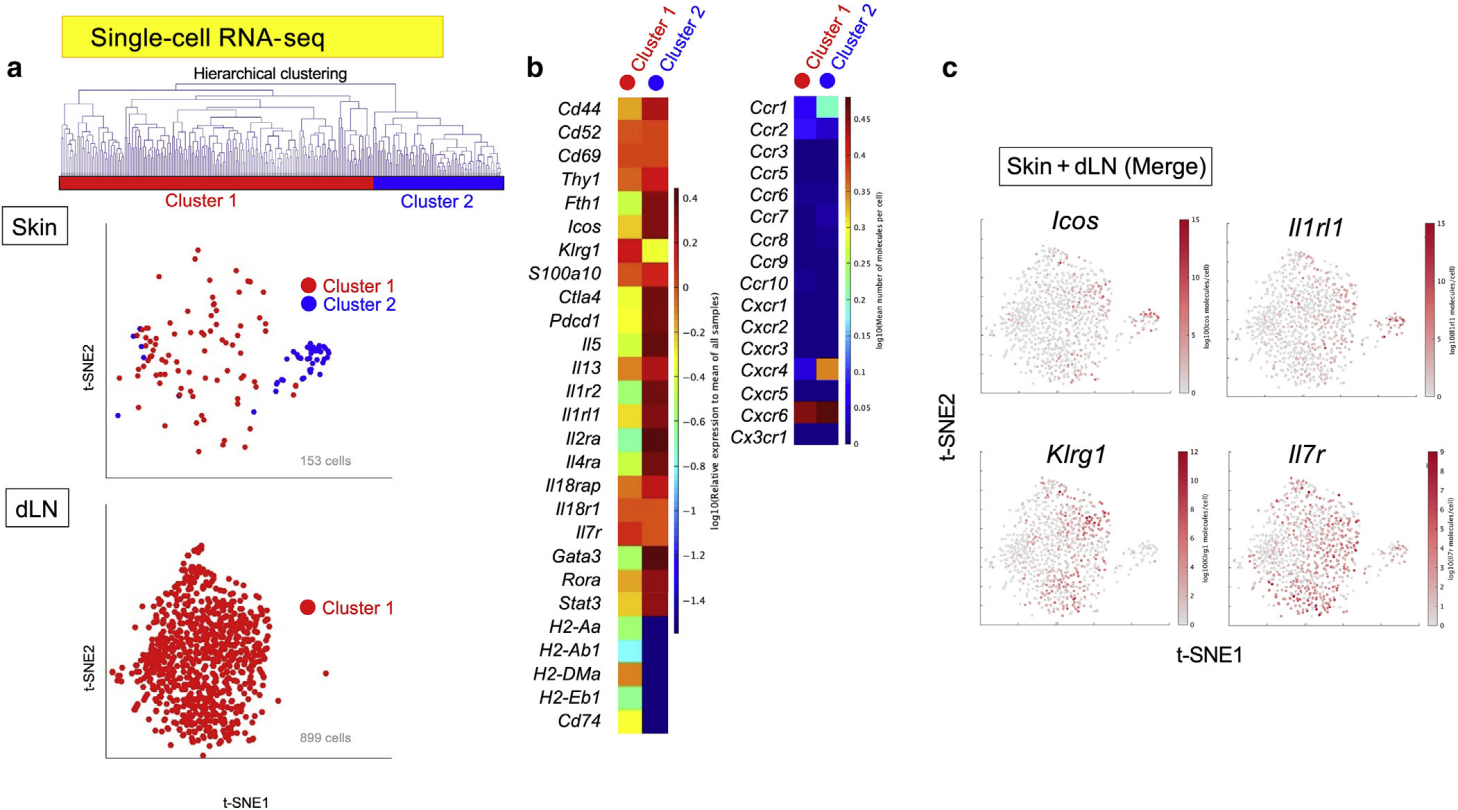
**Single-cell RNA-seq analysis of sorted ILC2s from the skin and the dLNs of transgenic mice expressing IL-33 (IL33tg).** (**a**) Single-cell RNA-seq identifies two clusters. t-SNE plot of the single-cell RNA-seq data of 1,052 ILC2s combined from three mice. Hierarchical clustering based on gene expression profiles was performed, and the ILC2s split into two clusters: cluster 1 (circulating ILC2s) and cluster 2 (skin-resident ILC2s). (**b**) Heatmaps of the representative ILC2-related genes and chemokine receptor‒related genes from each cluster. (**c**) Single-gene expression t-SNE plots of the single-cell RNA-seq data. Note that *Icos*, *Il1rl1*, *Klrg1*, and *Il7r* are markers of ILC2s. Representative data are from two independent experiments. dLN, draining lymph node; *Il1rl1*, IL-33 receptor alpha chain; ILC2, group 2 innate lymphoid cells; RNA-seq, RNA sequencing; t-SNE, t-distributed stochastic neighbor embedding.

We previously reported that the ILC2s were increased in the skin lesions and dLNs from the IL33tg mice compared with that in the wild-type mice (Imai et al., 2013). To resolve the molecular signatures of the skin ILC2s, we sorted the ILC2s from the skin lesions and dLNs of IL33tg mice and analyzed their transcriptomes using the BD Rhapsody Single-Cell Analysis System (BD Biosciences, San Jose, CA) for single-cell RNA sequencing (RNA-seq) analysis (Figure 1). Using hierarchical clustering, a cluster analysis method that attempts to build a hierarchy of clusters, we found that the skin ILC2s had split into two clusters: clusters 1 and 2 (Figure 1a). Conversely, only one cluster of lymph node ILC2s was found (cluster 1). Therefore, we hypothesized that among the ILC2s in the skin, only cluster 1 migrated to the dLNs, and cluster 2 remained in the skin. We named cluster 1, found in both the skin and lymph nodes, circulating ILC2s and named cluster 2, found only in the skin, skin-resident ILC2s. Gene-generated heatmaps revealed differences in the gene signatures expressed in each cluster (Figure 1b). Both the circulating ILC2s (cluster 1) and the skin-resident ILC2s (cluster 2) expressed *Ico*s and *Cd69*. However, both cell populations demonstrated minimal expression of chemokine receptor‒related genes (Figure 1b, right panel), consistent with the findings of Moro et al. (2016). The circulating ILC2s (cluster 1) expressed *Klrg1*, *Il7r*, and H2-related major histocompatibility complex (MHC) class II genes, including *Cd74*. Conversely, in the skin-resident ILC2s (cluster 2), *Il5*, *Il13*, and *Il2ra* were observed, and the expression of MHC class II‒related genes was low. The gene expression of the IL-33 receptor alpha chain was high in both clusters 1 and 2 but higher in cluster 2 (Figure 1c). Thus, we found that the skin ILC2s had split into two clusters.

### Development of IL33tg–KikGR mice

**Figure 2:**
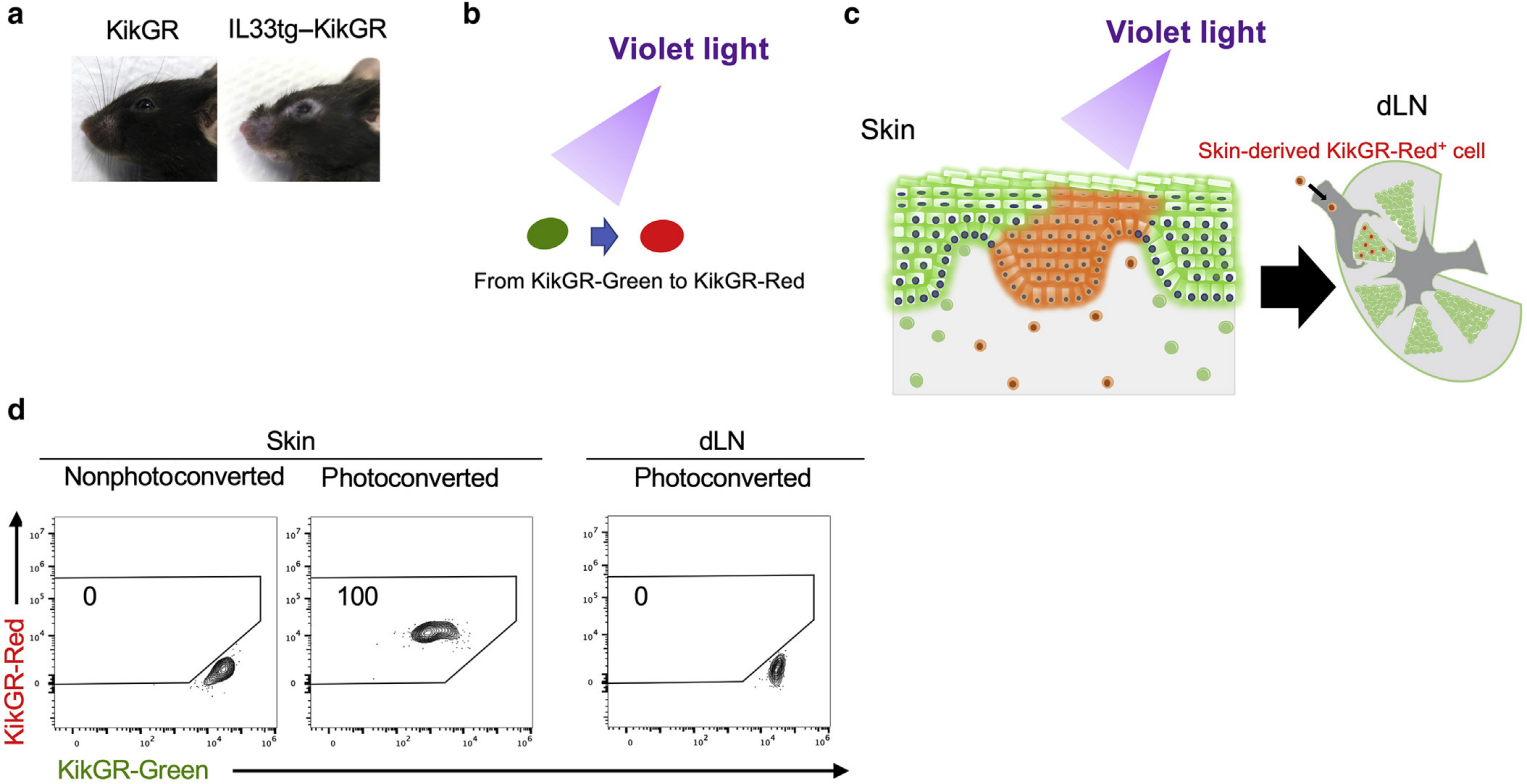
**Features of transgenic mice expressing IL-33 (IL33tg)– KikGR photoconvertible fluorescent protein.** (**a**) Cutaneous manifestations of the IL33tg–KikGR mice. (**b**) The concept of photoconversion. The KikGR photoconvertible fluorescent protein changes irreversibly to red (KikGR-Red) on exposure to violet light. (**c**) Conceptual figure of the experimental system. KikGR-Red cells were observed in the dLN after photoconversion, and these KikGR-Red cells can be distinguished from the KikGR-Green cells. (**d**) The IL33tg–KikGR mice were killed immediately after skin photoconversion, and the cells from their skin and dLNs were isolated for analysis by flow cytometry. We used nonphotoconverted mice as the control. At this point, the cells in violet light‒exposed skin were KikGR-Red, and the cells in the dLNs remained KikGR-Green. dLN, draining lymph node; KikGR, Kikume Green-Red.

It was an established theory that ILC2s do not migrate from the tissues in the steady state (Imai, 2019; Moro et al., 2016); therefore, we planned experiments to confirm whether skin ILC2s indeed migrate from the skin to the dLNs. We tracked the migration of ILC2s by crossing over IL33tg mice with KikGR knock-in mice (Tomura et al., 2014) to generate IL33tg–KikGR mice. AD-like skin lesions spontaneously developed in the IL33tg–KikGR mice, similar to those in the IL33tg mice (Figure 2a). KikGR, a photoconvertible fluorescent protein, changes color from green to red on exposure to violet light (Figure 2b). Exposing the inflamed skin of the IL33tg–KikGR mice to violet light allowed us to label the ILC2s in the skin and track their migration from the skin to the dLNs (Figure 2c). The mice were killed immediately after the experiment, regardless of whether their skin was photoconverted or nonphotoconverted. As displayed in Figure 2d, violet light caused the skin cells to be marked with KikGR-Red^+^ (100%). Conversely, the cells in the dLNs remained KikGR-Green^+^ (KikGR-Red^+^ cells: 0%) (Figure 2d, right panel).

### Migration of ILC2s from the skin to the dLNs under conditions of dermatitis

**Figure 3:**
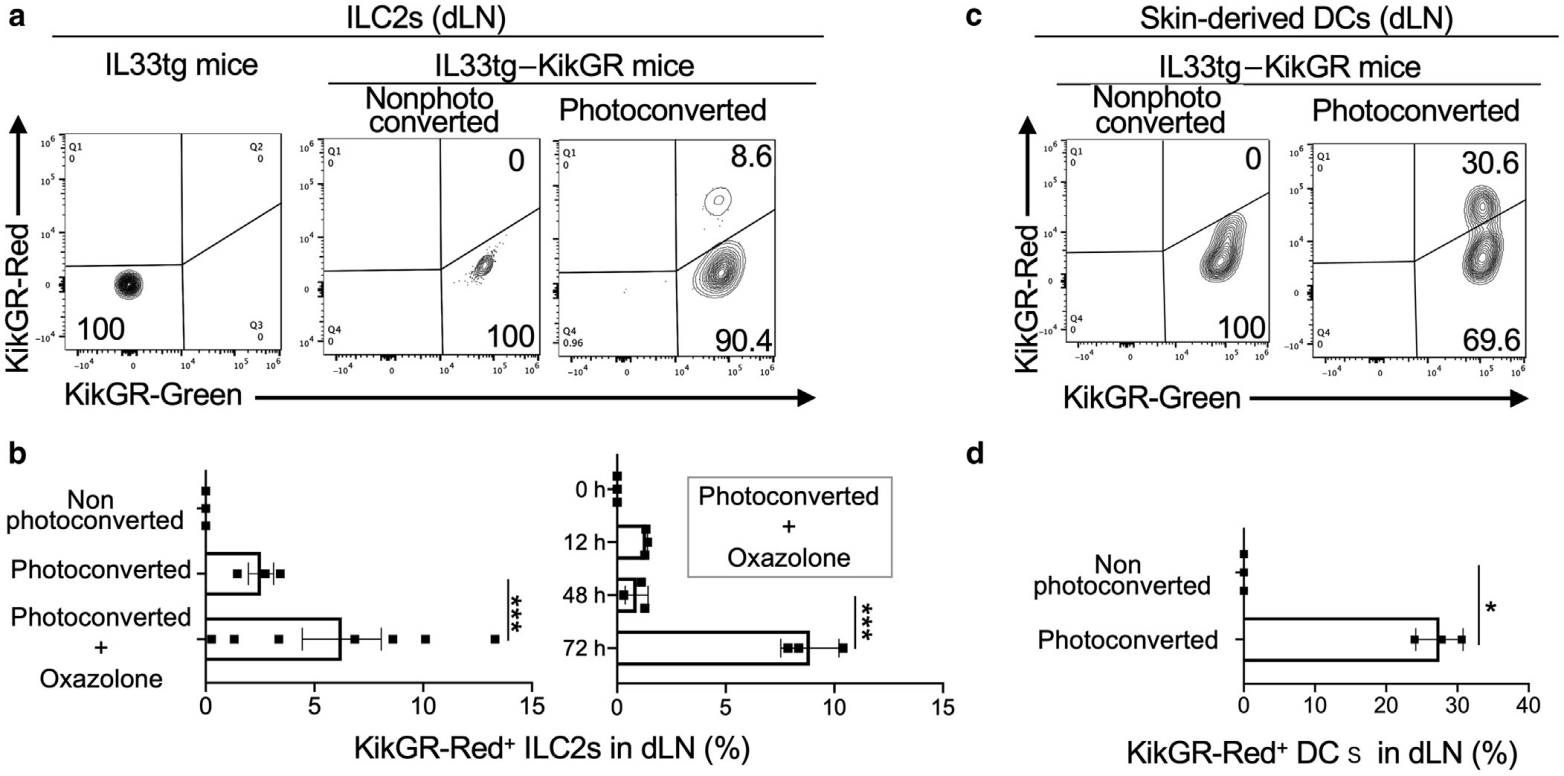
**ILC2s migrate from the skin to dLNs.** (**a**) Flow cytometry plots displaying KikGR-Green^+^ and KikGR-Red^+^ ILC2s in the dLNs 72 hours after skin photoconversion from the transgenic mice expressing IL-33 (IL33tg) or the IL33tg–KikGR mice. Cervical dLN cells were gated on ILC2s (see Figure 7a for the gating strategy). The top right number (8.6%) indicates the proportion of ILC2s that migrated from the skin, which were KikGR-Red^+^. The data are representative of three mice and three independent experiments. (**b**) Cells from the dLNs were stained to analyze the skin-derived ILC2s by flow cytometry 0–72 h (right panel) or 72 h (left panel) after skin photoconversion. The data represent the proportion of skin-derived ILC2s in the dLNs (labeled KikGR-Red) from the IL33tg–KikGR mice (n = 3–7). (**c**) Flow cytometry plots displaying the KikGR-Green^+^ and KikGR-Red^+^ skin-derived DCs in the dLNs 72 h after skin photoconversion from IL33tg–KikGR mice. Cervical dLN cells were gated on skin-derived DCs (see Figure 7b for the gating strategy). (**d**) Cells from the dLNs were stained to analyze the skin-derived DCs by flow cytometry 72 h after skin photoconversion. The data represent the proportion of skin-derived DCs in the dLNs labeled KikGR-Red from the IL33tg–KikGR mice (n = 3). Unpaired *t*-test was used to assess the statistical significance. ∗∗∗*P* < 0.001, ∗*P* < 0.05. Each dot represents a value for each mouse. The bold lines represent the estimated mean values, and the thin lines indicate the SEM values. DC, dendritic cell; dLN, draining lymph node; h, hour; ILC2, group 2 innate lymphoid cell; KikGR, Kikume Green-Red.

To test the hypothesis that migratory ILC2s exist in dLNs, we exposed the facial lesional skin of IL33tg–KikGR mice to violet light and photoconverted it as described in the Materials and Methods section, changing the cellular fluorescence from KikGR-Green to KikGR-Red. Using flow cytometry, we tested the KikGR-Green^+^ and KikGR-Red^+^ ILC2s within the dLNs 72 hours after the photoconversion of the skin (Figure 3a and b). Interestingly, we detected the skin-derived KikGR-Red^+^ ILC2s in the dLNs (Figure 3a). In addition, topical oxazolone increased the migration of ILC2s from the skin to the lymph nodes (Figure 3b, left panel). At 48 hours, the ILC2s had not yet migrated to the lymph nodes, and migration required 72 hours (Figure 3b, right panel) under the condition of topical oxazolone. Because we previously reported that skin-derived dendritic cells (DCs) were detectable in dLNs using the KikGR system in vivo (Tomura et al., 2014), we used DCs as a positive control (Figure 3c and d). Although slightly less frequent than skin-derived DCs, migration of skin ILC2s to the dLNs was confirmed at a frequency comparable with that of DCs.

### Gene signature sharing by lymph node- and skin-derived ILC2s in lymph nodes

**Figure 4:**
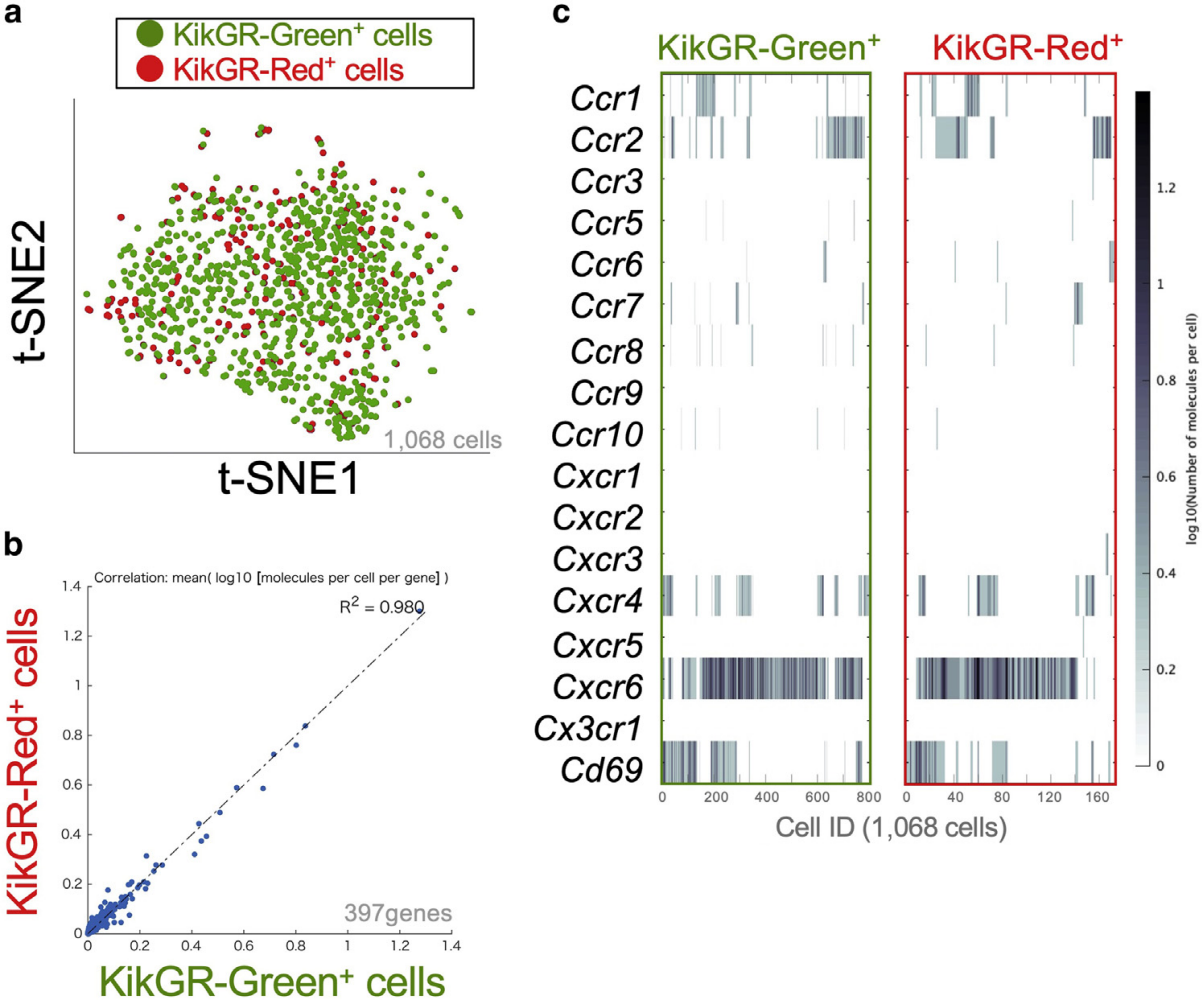
**Differential expression of genes of sorted lymph node-derived ILC2s KikGR-Green^+^ and skin-derived ILC2s KikGR-Red^+^ in lymph nodes 72 hours after skin photoconversion from transgenic mice expressing IL-33 (IL33tg)–KikGR.** (**a**) t-SNE plot of the single-cell RNA sequencing data of 1,068 KikGR-Green^+^ and KikGR-Red^+^ ILC2s. (**b**) Correlation plot for the log10 mean gene expression levels in the KikGR-Green^+^ ILC2s versus that in KikGR-Red^+^ ILC2s. R is the correlation coefficient obtained by Pearson’s product-moment correlation test (R^2^ = 0.980). Note that the KikGR-Green^+^ ILC2s and KikGR-Red^+^ cells shared the same gene signature. (**c**) Heat map displaying the level of expression of each chemokine receptor‒related gene in each cell. Cells are ordered by hierarchical clustering. Representative data are from two independent experiments. ID, identification; ILC2, group 2 innate lymphoid cell; KikGR, Kikume Green-Red; t-SNE, t-distributed stochastic neighbor embedding.

To resolve the molecular signatures of the lymph node- and skin-derived ILC2s in dLNs, we sorted the KikGR-Green^+^(Red^‒^) and KikGR-Red^+^ ILC2s from the dLNs of IL33tg–KikGR mice (e.g., Figure 3a, right panel; 90.4% for KikGR-Green^+^[Red^‒^] and 8.6% for KikGR-Red^+^) and analyzed their transcriptomes using single-cell RNA-seq analysis. The single-cell RNA-seq demonstrated that the lymph node-derived KikGR-Green^+^(Red^‒^) cells and the skin-derived KikGR-Red^+^ cells shared the same gene signature (Figure 4a–c). Therefore, we established that the circulating ILC2s, which had the same RNA gene expression, had migrated from the skin to the dLNs. We thus conclude that skin ILC2s consist of circulating and skin-resident ILC2s, whereas dLN ILC2s consist of only circulating ILC2s.

### Distinction of circulating and skin-resident ILC2s through flow cytometry and/or immunohistochemistry

**Figure 5:**
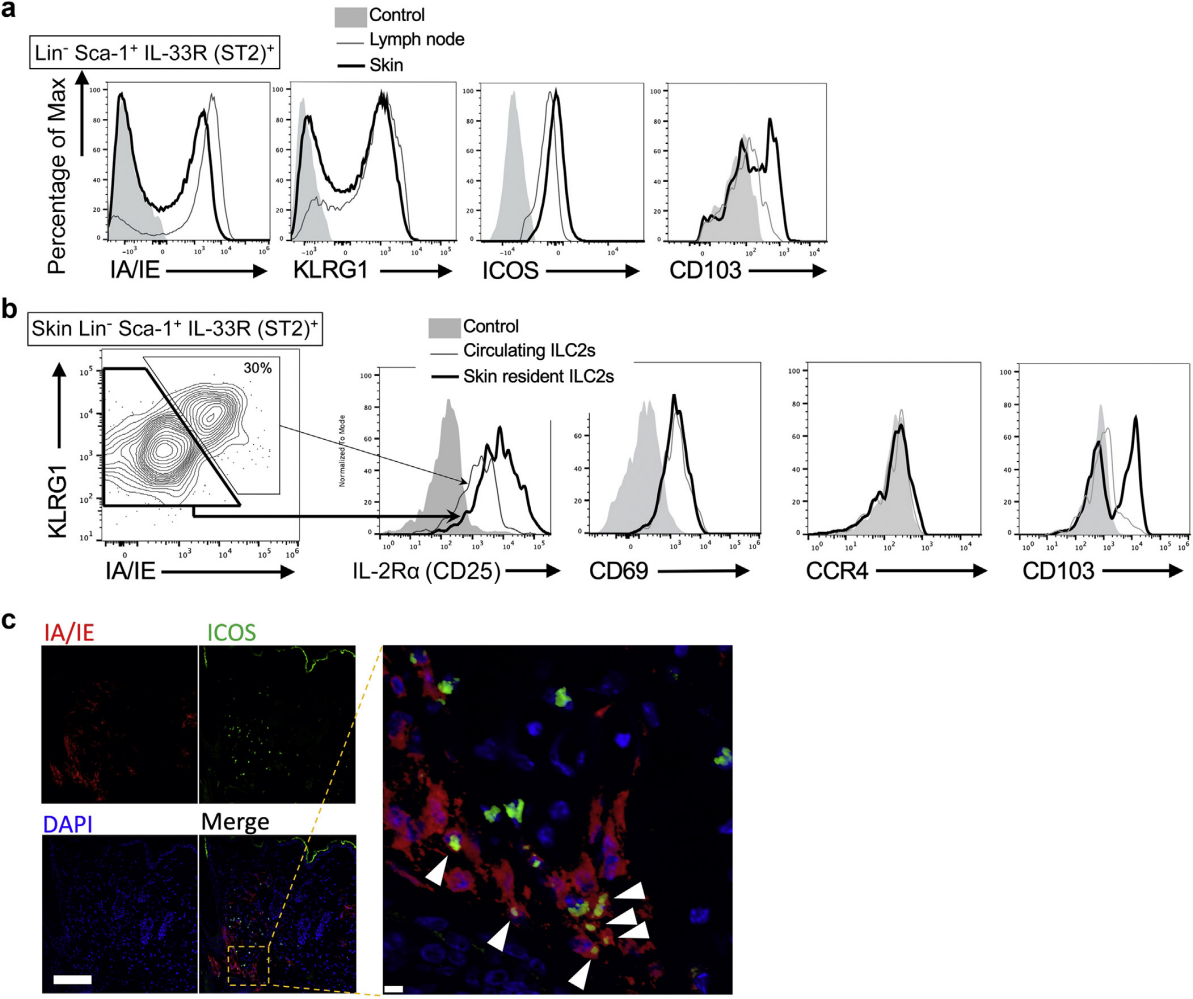
**Features of circulating ILC2s and skin-resident ILC2s.** (**a**) Flow cytometry of ILC2s from the lymph node (thin line) and skin (bold line) of IL33tg mice. Cells were gated on lineage marker (Lin)^−^ Sca-1^+^ IL-33R (ST2)^+^ cells for ILC2s. (**b**) Flow cytometry of cells from the skin of IL33tg mice. The cells were gated on an ILC2 fraction. Note that the ICOS^+^ KLRG1^+^ IA/IE^+^ cells are circulating ILC2s, and the ICOS^+^ KLRG1^‒^ IA/IE^‒^ cells are skin-resident ILC2s. (**c**) Immunofluorescence of IA/IE and ICOS in Rag2 knockout IL33tg mouse skin. Note that the ICOS^+^ IA/IE^+^ cells (red/green double‒positive cells, arrowheads) are circulating ILC2s, and the ICOS^+^ IA/IE^‒^ cells (green single‒positive cells) are skin-resident ILC2s. Representative data are from three independent experiments. Bar = 100 μm (inset, 10 μm). ILC2, group 2 innate lymphoid cell; Max, maximum.

Using flow cytometry, we investigated whether the gene expression of ILC2s matches surface marker expression. As expected, the ILC2s in the skin of the IL33tg mouse split into two populations (Figure 5a, thick line). The expression of KLRG1 and MHC class II was high on the cell surface of the circulating ILC2s and low on the cell surface of the skin-resident ILC2s (Figure 5b). Consistent with the gene expression (Figure 1b), the surface marker expression of the IL-2Rα (CD25) was higher in the skin-resident ILC2s than in the circulating ILC2s. Both the circulating ILC2s and the skin-resident ILC2s expressed CD69. Thus, the results obtained by single-cell RNA-seq were reproduced by flow cytometry. Integrins such as CD103 are known to be involved in cell migration (Mackay et al., 2013). Interestingly, only skin-resident ILC2s express CD103 (Figure 5b, the rightmost panel), suggesting that CD103 is a marker for skin-resident ILC2.

Next, to investigate how skin-resident and circulating ILC2s are distributed in the skin, the histology of dermatitis was examined using immunohistochemistry (Figure 5c). Because there are no specific markers in ILC2, it is difficult to identify ILC2s through immunohistochemistry. To address this problem, we previously reported that ICOS^+^ cells correspond to ILC2s in Rag2 knockout IL33tg mouse skin because both ILC2s and T cells express ICOS, but Rag2 knockout mice lack T cells (Imai et al., 2019). We confirmed through immunohistochemistry that similar to the flow cytometry results, the ICOS^+^ IA/IE^+^ cells were circulating ILC2s, and the ICOS^+^ IA/IE^‒^ cells were skin-resident ILC2s (Figure 5c). However, both were distributed mainly in the dermis, and neither of them had a characteristic distribution.

### Production of large numbers of cytokines by skin-resident ILC2s

**Figure 6:**
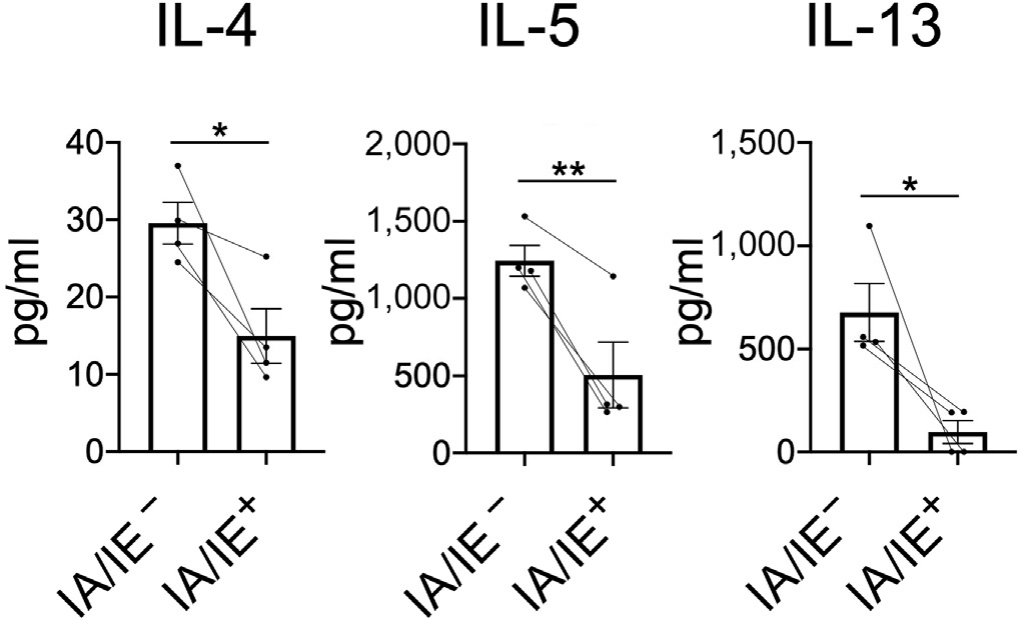
**Skin-resident ILC2s (IA/IE**^**‒**^**) produce large numbers of cytokines.** Type 2 cytokine production by the sorted ILC2s (IA/IE^+^ or IA/IE^‒^ population; 2 × 10^4^ cells per well) from the skin of transgenic mice expressing IL-33 (IL33tg mice) were cultured 24 hours after stimulation with phorbol myristate acetate/ionomycin. The type 2 cytokines (IL-4, IL-5, IL-13) in each supernatant were measured using ELISA (n = 4). Paired *t*-test was used to assess the statistical significance. ∗∗*P* < 0.01; ∗*P* < 0.05. ILC2, group 2 innate lymphoid cell.

Finally, using ELISA, we investigated whether the gene expression of type 2 cytokines in skin-resident ILC2s (Figure 1b, cluster 2) matches the protein expression. We sorted the IA/IE^+^ circulating ILC2s and IA/IE^‒^ skin-resident ILC2s from the lesional skin of IL33tg mice and cultured and stimulated them with phorbol myristate acetate/ionomycin. We then measured the type 2 cytokines in each supernatant using ELISA. Skin-resident ILC2s, which were IA/IE negative, were revealed to be highly capable of producing IL-4, IL-5, and IL-13 (Figure 6).

## Discussion

Because the cutaneous ILC2s in wild-type mice are very few and difficult to analyze (Imai et al., 2013), we used IL33tg mice, which facilitated our study of ILC2s. In this study, through single-cell RNA-seq analysis, we found that the ILC2s in the skin of the IL33tg mice split into two clusters. Although most studies perform single-cell transcriptomic analysis of all cell types, rare cells such as ILC2 are too few to be reflected in the analysis results (Rojahn et al., 2020). To address this shortcoming, in this study, we investigated isolated ILC2s before performing single-cell RNA-seq analysis. Recently, the tissue-specific heterogeneity of ILC2s in the bone marrow and peripheral tissues (lung, gut, fat, skin) was reported, as determined using single-cell RNA-seq analysis (Ricardo-Gonzalez et al., 2018; Schneider et al., 2019). However, no study has yet tried to classify cutaneous ILC2 into subgroups. ILC2s are believed to be tissue-resident cells under steady-state conditions (Dutton et al., 2019; Moro et al., 2016; Schneider et al., 2019). However, it occurred to us that ILC2s may migrate rather than merely remain in the local skin because ILC2 in the peripheral blood has been reported to affect the pathogenesis of AD in humans (Imai et al., 2021). The migration of ILC2 has not been investigated in inflammatory conditions of dermatitis caused by excess IL-33. In this study, using KikGR‒IL33tg mice, we found that circulating ILC2s migrate at least between the skin and the dLNs, whereas skin-resident ILC2s settle in the skin. Recently, it has been reported (Ricardo-Gonzalez et al., 2020) that using the helminth infection system, the activation of local tissue ILC2s by tissue-specific alarmins (Oppenheim and Yang, 2005) can induce the lymph node migration and blood dissemination of ILC2s from the gut and lung, resulting in the systemic distribution of type 2 cytokines. In this study, we showed for the first time that the ILC2s in the skin migrate to the lymph nodes when activated by cutaneous alarmin IL-33. Together with the results of our study, these results suggest that the activated local ILC2s are extruded from the local tissue into the lymph nodes, changing the local natural response to a systemic type 2 response.

The role of ILC2s in regulating antigen presentation and T-cell activation was recently proposed (McKenzie et al., 2014). ILC2s can present antigens to the T cells through the expression of MHC II, and this T helper type 2 activation occurs in an MHC class II‒dependent manner. In addition to this dialog between the ILC2s and T cells, ILC2s may also affect the initiation of adaptive T helper 2 cellular responses by altering the DC function (Halim et al., 2016). Therefore, our results suggest that circulating ILC2s play an important role in inducing adaptive immune responses by migrating from the skin to the dLNs. Conversely, the skin-resident ILC2s produced large numbers of type 2 cytokines, and we found that this population did not migrate to the lymph nodes, as previously reported (Kobayashi et al., 2020; Moro et al., 2016).

Several limitations need to be considered. Because single-cell RNA-seq analysis is a novel method, there is no consensus on a standard bioinformatics approach. In this study, we used established ILC2 marker genes and analyzed data using the BD DataView software, version 1.2.2 (BD Biosciences), with the default setting, but a more useful calculation method can soon become mainstream. We have previously shown that the ILC2s infiltrating the lesional skin of IL33tg mice are mainly present in the dermis (Imai et al., 2019). In this study, both circulating and skin-resident ILC2s were distributed mainly in the dermis, but we could not find any difference in the distribution of these two cell populations (Figure 5c). This, therefore, needs to be further investigated. Recently, it has been reported that ILC2s are CXCR6 positive and can migrate in response to CXCL16 (Li et al., 2019). We comprehensively analyzed the expression of chemokine receptors in circulating and skin-resident ILC2s and found almost no difference (Figure 1b). Therefore, the mechanism by which ILC2s migrate needs to be further investigated. CD103s are well-known markers of skin-resident T cells (Mackay et al., 2013), and we showed that skin-resident ILC2s expressed higher expression of CD103 than circulating ILC2s (Figure 5b). Therefore, skin-resident ILC2 may remain in the skin by a mechanism similar to that by which skin-resident T cells remain in the skin.

In conclusion, we found that the skin ILC2s split into two clusters: circulating and skin-resident ILC2s. As summarized in Table 1, the MHC II‒positive circulating ILC2s could be involved in adaptive immune responses by migrating from the skin to the dLNs and could act as systemic arbiters of type 2 immunity, as previously reported (McKenzie et al., 2014). In contrast, the skin-resident ILC2s produced large numbers of type 2 cytokines at the local site and did not migrate to the lymph nodes. Thus, ILC2s may be crucial for innate immunity and for the induction of adaptive immune responses.

**Table 1:** Summary of Functions and Features of Circulating and Skin-Resident ILC2s

Subtype	Markers	Functions/Features
Circulating ILC2	ICOS KLRG1 MHC class II	Induces adaptive immune responses by migrating from the skin to the draining lymph nodes
Skin-resident ILC2	ICOS IL-2Rα (CD25) CD103	Produces a large amount of type 2 cytokines Tissue resident

Abbreviations: ILC2, group 2 innate lymphoid cell; MHC, major histocompatibility complex.

## Materials and Methods

### Mice

The mouse line hK14mIL33tg (IL33tg) expressing IL-33 driven by a keratin-14 promoter was previously described (Imai et al., 2019, 2017, 2013). The Rag2 knockout mice were from Taconic Biosciences (Germantown, PA). The KikGR mice, established with KikGR expression in all the cells of the mice, were previously described (Tomura et al., 2014). IL33tg mice were crossed over with KikGR mice to generate IL33tg–KikGR mice. All the animal studies were reviewed and approved by the Animal Use and Care Committee of Hyogo College of Medicine (Nishinomiya, Japan) and were designed per the International Guiding Principles for Biomedical Research Involving Animals published by the Council for International Organizations of Medical Sciences (Geneva, Switzerland). All the mice that were used in this study were maintained under specific pathogen-free conditions.

### Photoconversion

Photoconversion of the skin of KikGR mice was performed as previously described (Tomura et al., 2014). Briefly, IL33tg–KikGR mice were anesthetized, and their ear/face skin (location of dermatitis) was exposed to violet light for 90 seconds (111 mW/cm^2^ from SUPERLITE I01 spot 405 nm LED curing equipment, LUMATEC, Deisenhofen, Germany). Violet light (405 nm) was used so it would not cause inflammation, and we previously showed that exposure to violet light (10 minutes) indeed did not induce inflammation (Tomura et al., 2014).

### Antibodies

The fluorescence-labeled antibodies for mice B220, CD3, CD4, CD8, CD16/32, CD25, CD45, CD278 (ICOS), Gr-1, NK1.1, Sca-1, KLRG1, IA/IE, and Brilliant Violet 421 streptavidin were from BioLegend (San Diego, CA). The anti‒Siglec-F antibody was from BD Biosciences, and the anti-FcεRI (MAR-1) antibody and biotinylated anti-ST2 (IL-33R) antibody (RMST2-33) were from eBioscience (San Diego, CA).

### Flow cytometry

**Figure 7:**
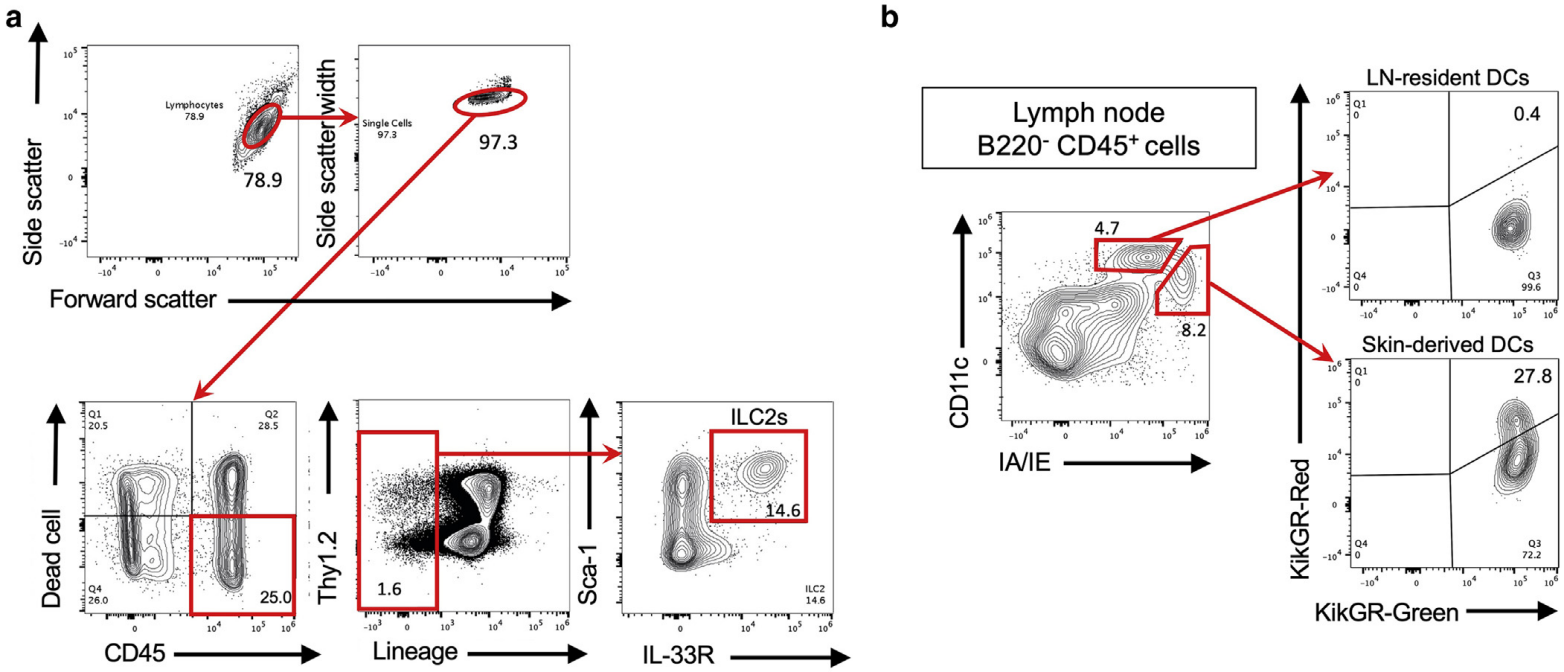
**Gating strategy for flow cytometry.** (**a**) Gating strategy for the analysis of FSC^low^SSC^low^CD45^+^ lineage (Lin)^−^Thy1.2^+^Sca-1^+^ IL-33R (ST2)^+^ ILC2s. The numbers indicate the percentage of cells in each gate. (**b**) Gating strategy for the analysis of CD45^+^ B220^−^ DCs within the draining LNs 72 hours after the photoconversion of the skin. The LN-resident DCs are CD11c^high^ IA/IE^med^, and the skin-derived DCs are CD11c^med^ IA/IE^high^. Note that only skin-derived DCs contain KikGR-Red^+^ cells. The numbers indicate the percentage of cells in each quadrant. Similar results were obtained from two independent experiments. DC, dendritic cell; ILC2, group 2 innate lymphoid cell; KikGR, Kikume Green-Red; LN, lymph node.

Skin specimens or dLNs were homogenized and incubated in an RPMI 1640 medium containing 1% fetal calf serum, 0.01% DNase I (Roche, Basel, Switzerland), and 21.25 μg/ml LiberaseTH (Roche) at 37 °C for 60 minutes. Dead cells were excluded by staining them with fixable viability dye APC-eFluor 780 (eBioscience). Cells were incubated with anti-CD16/32 (FcR III and FcR II) antibody for blocking before staining with each antibody. The stained cells were analyzed through flow cytometry using an SP6800 Spectral Cell Analyzer (Sony Biotechnology, Tokyo, Japan). The data were analyzed using the FlowJo software, version 10.7 (Tree Star, Ashland, OR). The classification of the ILC2s was lineage markers (Lin) (B220, CD3, CD4, CD8, Gr-1, FcεRI, NK1.1, Siglec-F)^–^ CD45^+^ Sca-1^+^ ST2^+^ cells. The precise gating strategies used to identify the ILC2s were previously described (Imai et al., 2019) and can be viewed in Figure 7a. The classification of the skin-derived DCs in the dLNs was B220^−^ CD45^+^ IA/IE^high^ CD11c^med^ cells. The precise gating strategies used to identify the skin-derived DCs were previously described (Tomura et al., 2014) and can be viewed in Figure 7b. In several experiments, the ILC2s were purified by electronic cell sorting using BD FACSAria III Cell Sorter (BD Biosciences).

### ELISA

The concentrations of cytokines and chemokines in the culture supernatants were measured using a Bio-Plex Protein Array System (Bio-Rad Laboratories, Hercules, CA) according to the manufacturer’s instructions.

### Tissue staining and immunofluorescence

Staining for ICOS and IA/IE was performed using frozen 20 μm sections of mouse skin. The sections were air dried after 20-minute fixation in cold acetone and blocked for 1 hour at room temperature with 5% goat/rat serum. They were then incubated with Alexa Fluor 488 anti-ICOS antibody (BioLegend) and Alexa Fluor 594 anti-mouse IA/IE antibody (BioLegend). After mounting with a ProLong Diamond Antifade with DAPI (Life Technologies, Gaithersburg, MD), fluorescence images were recorded using an LSM780 confocal laser scanning microscope (Carl Zeiss Microscopy, Thornwood, NY).

### Single-cell RNA-seq analysis

ILC2s were isolated from mouse skin or dLN samples using BD FACSAria III Cell Sorter. Targeted single-cell RNA-seq analysis was then performed using the BD Rhapsody Single-Cell Analysis System according to the manufacturer’s instructions. For the library construction, we used the BD Mouse Immune Single-Cell Multiplexing Kit (#633793, BD Biosciences) and the BD Rhapsody Immune Response‒Targeted Panel for Mouse (#633753, BD Biosciences), which consisted of primer sets for 397 genes. Sequencing was performed using an Illumina HiSeq X (Illumina. San Diego, CA). The fastq files were converted by the cloud software using BD Rhapsody Analysis Pipeline (BD Biosciences) and then analyzed using the BD DataView software, version 1.2.2. Hierarchical clustering and differential expression were performed in the BD DataView software using the built-in cluster function of MATLAB (R2014a, MathWorks, Natick, MA) (Joshi et al., 2017; Zhang et al., 2018) according to the manufacturer’s instructions (BD Rhapsody Handbook Rev. 3.0).

### Statistical analyses

Statistical analyses were performed using GraphPad Prism 8 (GraphPad Software, San Diego, CA). Significance was set at *P* < 0.05.

### Data availability statement

Datasets related to single-cell RNA sequencing can be found at https://www.ncbi.nlm.nih.gov/sra/PRJNA719176, hosted at National Center for Biotechnology Information's Sequence Read Archive database.

## ORCIDs

Minori Nakatani-Kusakabe: https://orcid.org/0000-0003-4422-4471

Koubun Yasuda: https://orcid.org/0000-0002-3533-1702

Michio Tomura: https://orcid.org/0000-0002-1419-6492

Makoto Nagai: https://orcid.org/0000-0003-3638-706X

Kiyofumi Yamanishi: http://orcid.org/0000-0003-0484-2320

Etsushi Kuroda: https://orcid.org/0000-0002-7198-2190

Nobuo Kanazawa: https://orcid.org/0000-0003-3000-9711

Yasutomo Imai: https://orcid.org/0000-0003-3169-5717

## Author Contributions

Conceptualization: YI; Data Curation: KYam, MN, MNK, YI; Formal Analysis: KYas, MNK, YI; Funding Acquisition: NM, YI; Investigation: MN, MNK, YI, KYas; Methodology: MT, YI; Project Administration: KYam, YI; Resources: MT, Supervision: EK, MT, NK; Validation: KYam, YI; Visualization: MNK, YI; Writing - Original Draft Preparation: MNK, YI; Writing - Review and Editing: YI
